# Failed surgical ligation of the proximal left subclavian artery during hybrid thoracic endovascular aortic repair successfully managed by percutaneous plug or coil occlusion: a report of 3 cases

**DOI:** 10.1186/1749-8090-6-45

**Published:** 2011-04-08

**Authors:** Geert Maleux, Johan Vaninbroukx, Sam Heye

**Affiliations:** 1 Department of Radiology, University Hospitals Leuven, Belgium

## Abstract

Open surgical rerouting and proximal ligation of one or more supra-aortic vessels prior to endovascular stent-graft placement has become an alternative to major open thoracic surgery in the treatment of complex thoracic aortic disease. Complications owing to failed surgical ligation of the left subclavian artery are rare. In this report, 3 cases of failed ligation are presented. Diagnosis was made by CT-scan and treatment was performed by transcatheter coil and plug embolization, avoiding redo neck surgery.

## Background

Endovascular repair has become a valuable alternative to open repair for the treatment of several thoracic aortic pathologies [1-4]. However, stent-graft placement requires an adequate proximal and distal landing zone in the aorta of at least 2 cm in order to avoid early or late type I endoleak. Therefore, surgical ligation and rerouting of one or more supra-aortic vessels can be necessary for safe stent-graft deployment and efficient and durable clinical outcome. Recent reports deal with the successful technical and clinical outcome after supra-aortic rerouting [5-7]. However, type and management of complications related to this type of open vascular surgery are scarce and not well-documented [7]. In this report we present the clinical and radiological outcome after endovascular management of failed surgical ligation of the left subclavian artery during supra-aortic rerouting for safe thoracic stent-graft placement.

From 1999 to end of 2009, 172 thoracic stent-graft procedures in 160 patients were performed in the author's institution. In 49 patients (30%), supra-aortic rerouting was performed. In 41 out of these 49 patients (84%) perioperative surgical ligation of the left subclavian artery was performed in association with supra-aortic rerouting. All patients were followed up according to the EUROSTAR guidelines [8]. In 3 out of these 41 patients (7%) previously treated by left subclavian artery ligation, persistent flow through the ligated artery was identified and associated with gradual increase of aneurismal or false luminal diameter. There were no patients with persistent retrograde flow through the prevertebral left subclavian artery, but with a stable or decreasing aneurismal sac.

## Case Presentation

### Case 1

**Figure 1:**
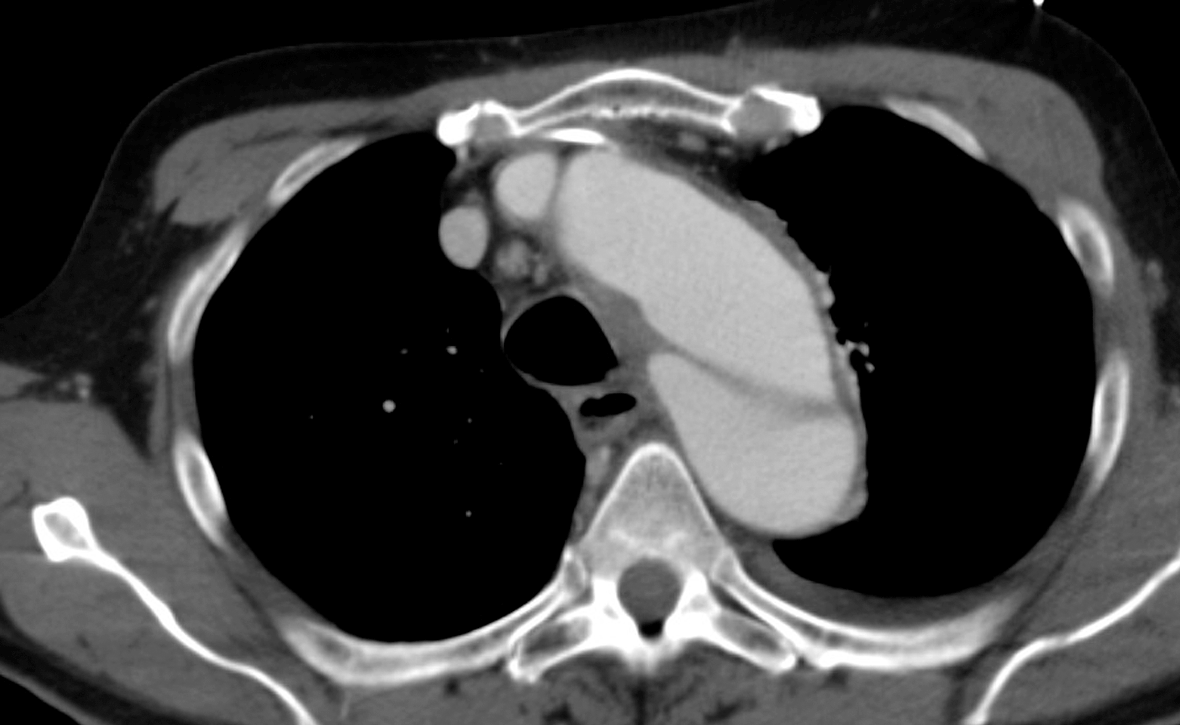
**Thoracic aortic CT-scan at admission reveals a classic Stanford type B aortic dissection without clear false lumen dilatation**.

**Figure 2:**
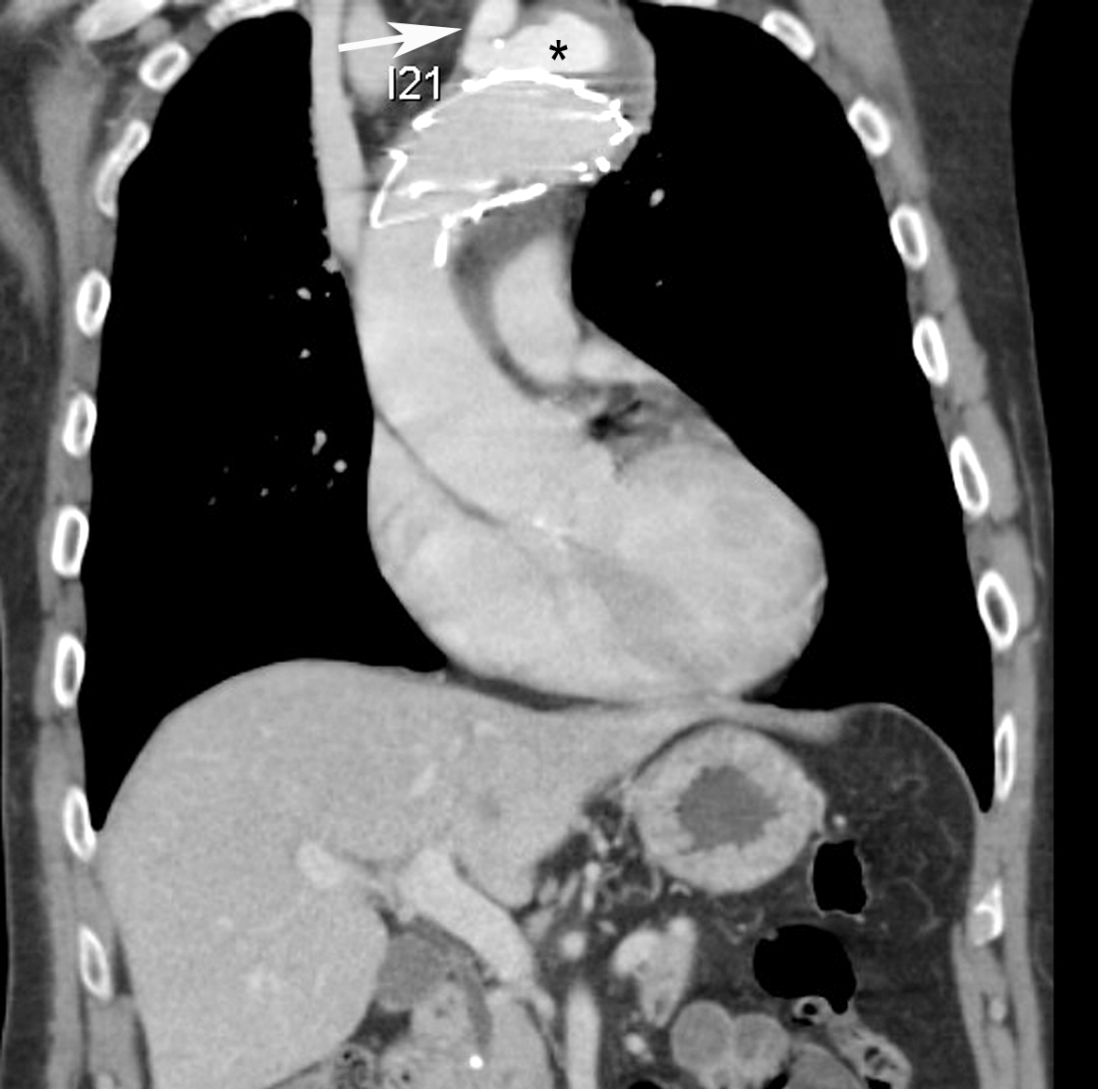
**Coronal CT-reconstruction image one year after stent-grafting shows persistent opacification of both the left subclavian artery (white arrow) and the false thoracic aortic lumen (black asterisk)**.

**Figure 3:**
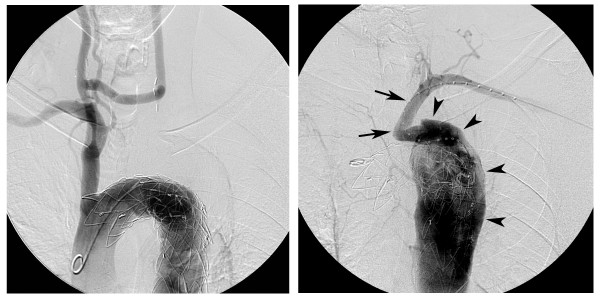
**Catheter angiography of the aortic arch**. (a) Catheter angiography of the aortic arch, 13 months after hybrid surgery shows the stent-graft in place, starting just distal to the origin of the brachiocephalic trunk. Note also the good patency of the carotido-carotid bypass. There is no opacification of the left subclavian artery. (b) After puncturing the left brachial artery, a calibrated pigtail is navigated through the proximal left subclavian artery (arrows) into the false lumen of the thoracic aorta (arrowheads). Note the antegrade flow in the left subclavian artery.

**Figure 4:**
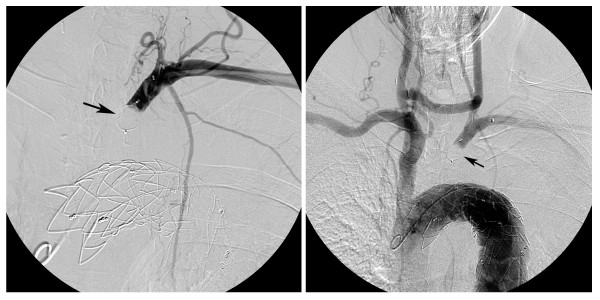
**Angiography after Amplatzer-plug deployment**. (a) Selective injection of contrast medium in the distal left subclavian artery after Amplatzer-plug deployment (arrow) demonstrates a total occlusion of the proximal left subclavian artery. (b) Flush aortography after Amplatzer-plug deployment (arrows) reveals retrograde opacification (subclavian steal phenomenon) of the left subclavian artery through the retrogradely filling left vertebral artery.

A 65-year-old man presented with persistent thoracic pain since two weeks. Serial computed tomography (CT) scans revealed an aortic dissection, Stanford type II starting at the origin of the left subclavian artery (Figure 1) and with progressive increase of thoracic aortic diameter up to 5 cm over a two week time period. A decision was taken to exclude the aneurismal false lumen with use of a stent-graft (Talent, Medtronic, Santa Rosa, CA, USA). Because of unintentional covering of the origin of the left common carotid artery, a carotidocarotid bypass was performed, but despite many intraoperative efforts, it was not possible to ligate the proximal left subclavian artery via the cervicotomy. The patient recovered well without neurological sequellae and the thoracic pain disappeared progressively. Control physical examination at 3, 6 and 12 months after the thoracic endovascular aortic repair (TEVAR) was uneventful except for a persistent left radial pulse. CT-scans revealed a persistent opacification of the false lumen and the entire left subclavian artery (Figure 2). A discrete increase in thoracic aortic diameter up to 59 mm was noted. Catheter angiography performed 13 months after initial EVAR showed a fully patent stent-graft and retrograde opacification of the false lumen through connections between true and false aortic lumen at the level of the thoraco-abdominal and abdominal aorta; no subclavian steal phenomenon was identified (Figure 3a-b). After puncturing the left brachial artery and cannulation of the proximal subclavian artery and false lumen, selective angiography revealed antegrade opacification of the left subclavian and vertebral artery: the left upper limb and left posterior circulation was feeded antegradely via the retrogradely perfused false aortic lumen. It was decided to occlude the prevertebral segment of the left subclavian artery using a 16 mm diameter vascular plug (Amplatzer plug, AGA Medical, Plymouth, MN, USA). Completion angiography after plug placement revealed a suclavian steal via retrograde opacification of the left vertebral artery and antegrade opacification of the subclavian artery with exception of the completely thrombosed prevertebral segment (Figure 4a-b). Clinically, there was no more radial pulse palpable and symptoms of left arm claudication were noted, but these were managed conservatively.

**Figure 5:**
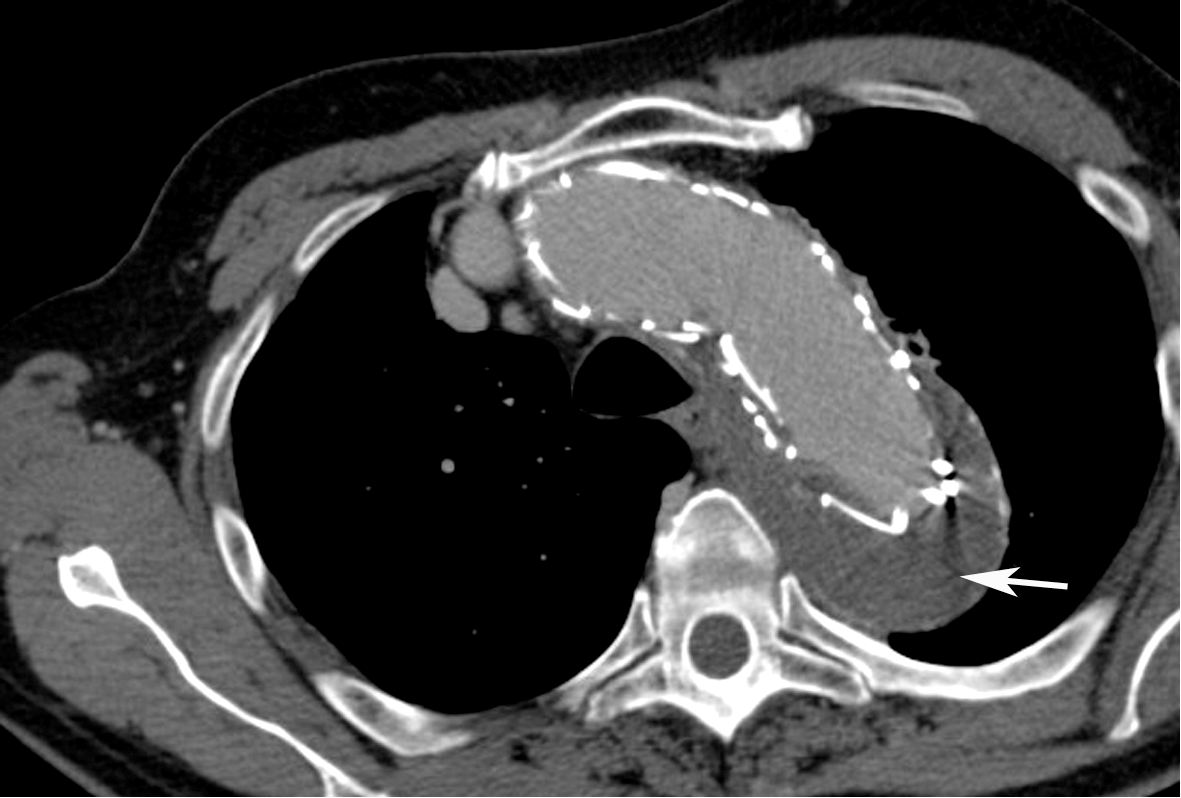
**Control CT-scan 3 years after stent-graft extension shows a stable thoracic aortic diameter of 50 mm without contrast opacification of the excluded false lumen, both in the early arterial and in the late venous phase**.

Control CT-scan one year later showed a progressive increase in diameter of the distal thoracic aorta below the stent-graft. An extension stent-graft (Talent, Medtronic, USA) was successfully placed landing at the tenth thoracic vertebra. The patient was discharged 3 days later. CT-scan at 1, 2 and 3 years follow-up after placement of the extention stent-graft revealed complete thrombosis of the false lumen and occlusion of the left subclavian artery with the occlusion-plug in place. The diameter of the thoracic aorta remained stable with a maximum diameter of 50 mm (Figure 5).

### Case 2

A 78-year-old man presented with an asymptomatic aneurysm of the proximal descending thoracic aorta with a maximal diameter of 66 mm. The patient already underwent endovascular exclusion of an abdominal aortic aneurysm two years earlier. It was decided to exclude the thoracic aneurysm with use of a stent-graft (Valiant, Medtronic, Santa Clara, CA, USA) after placing a carotidosubclavian bypass and ligation of the proximal left subclavian artery in order to minimize potential postoperative neurological symptoms related to myelum ischemia. The postoperative period was uneventful except for fever up to 38°C for 3 days; no signs of arm claudication were noted. Control CT-scan 6 months later revealed discrete increase of the aneurismal sac diameter up to 69 mm owing to a type II endoleak by retrograde sac perfusion through the incompletely ligated proximal left subclavian artery. It was decided to treat the endoleak. Under local anesthesia, the left brachial artery was punctured and a 45 cm long 8 F sheath (Arrows, Reading, PE, USA) was inserted. Angiography revealed the retrograde opacification of the prevertebral segment of the left subclavian artery, resulting in a type II endoleak. A 16 mm nominal diameter vascular plug (Amplatzer vascular plug, AGA Medical, Plymouth, MN, USA) was placed at the origin of the left subclavian artery, with complete disappearence of the endoleak. Control CT-scan at one and two years follow-up revealed absence of any residual type II endoleak and stable diameter of the thoracic aneurysm up to 68 mm.

### Case 3

**Figure 6:**
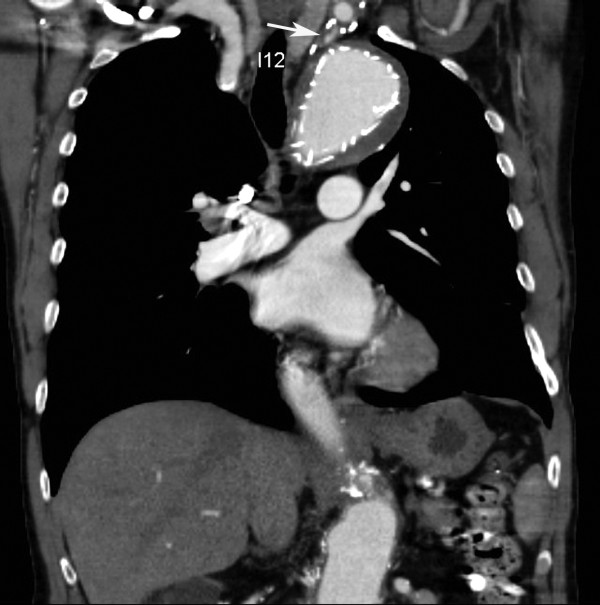
**Coronal CT-reconstruction image 6 months after stent-grafting reveals a faint opacification (white arrow) of the proximal left subclavian artery with focal opacification of the aneurismal sac lumen**.

**Figure 7:**
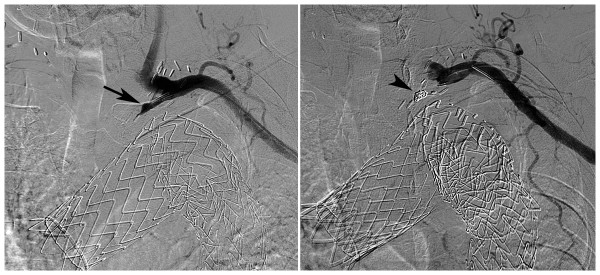
**Catheter angiography of the left subclavian artery**. (a) Selective injection of the left subclavian artery through a brachial artery catheter shows a well-functioning carotido-subclavian bypass and faint retrograde opacification of the proximal left subclavian artery and aneurismal sac (arrow), suggesting a type II endoleak. (b) After coil embolisation (arrowhead), there is no more opacification of both the proximal left subclavian artery and the small type II endoleak.

A 68-year-old man presented with an asymptomatic, focal atherosclerotic aneurysm of the aortic arch (maximal diameter of 6 cm) and another, focal, thoraco-abdominal aneurysm with a diameter of 5.5 cm, ending at the level of the origin of the renal arteries. Eleven years ago, the patient also underwent an elective surgical repair for an infrarenal abdominal aortic aneurysm. It was decided to first treat the arch aneurysm with use of a hybrid vascular procedure: A carotid-carotid bypass with additional bypass to the left subclavian artery was performed using a Silver 8 mm vascular graft; concomitantly a surgical ligation of the left subclavian artery proximal to the origin of the left vertebral artery was performed. Afterwards a stent-graft (TAG, W.L. Gore & Associates, Flagstaff, AZ, USA) starting at the origin of the brachiocephalic trunk and ending in the descending thoracic aorta, was inserted and resulting in a complete exclusion of the aneurysm. Postoperative follow-up was uneventful and three months later, patient underwent a Crawford operation for his thoraco-abdominal aneurysm with reimplantation of all visceral arteries including celiac trunk, superior mesenteric artery and both renal arteries. Six months later, follow-up CT-scan revealed a growing thoracic arch aneurysm and a type II endoleak by retrograde perfusion of the aneurysmal sac through an incompletely ligated left subclavian artery (Figure 6). It was decided to treat the type II endoleak by transcatheter technique. After local anesthesia, the left brachial artery was punctured and a 4F sheath was introduced. Through a 4F Cobra-catheter (Cook Medical, Bloomington IN, USA) a microcatheter (Miraflex, Cook Medical, Bloomington IN, USA) was navigated with the tip in the proximal left subclavian artery. Deployment of 3 fibered microcoils (Target Therapeutics, Boston Scientific Corporation, Natick, MA, USA) completely occluded the origin of the left subclavian artery with disappearance of the endoleak (Figure 7a-b). Control CT-scan 9 months later revealed a completely excluded thoracic aortic aneurysm without endoleak and stable in diameter.

## Discussion

Combined open and endovascular surgical repair is recently propagated as a less invasive treatment option for the management of aortic arch pathologies like aneurysms, dissections or penetrating ulcers [1,7,9-12]. However, these operations are also not free of early or late complications: myocardial infarction, respiratory and renal failure, postoperative hematoma, vertebrobasilar insufficiency or stroke are potential complications [13-18]. In this study we report on a yet unreported, not very uncommon (7% of all supraaortic rerouting cases with ligation), but silent complication after supra-aortic rerouting, namely an incomplete ligation of the left subclavian artery resulting in persistent perfusion of the thoracic aneurysm in two cases and in persistent, retrograde perfusion of the false lumen in the remaining case. Additionally, in all cases these radiological findings were associated with a gradual growth of the aneurismal sac or false lumen, stressing the importance of this silent complication. Adequate treatment seems to be mandatory to avoid potential late rupture. In the presented cases, a surgical attempt was made to ligate the prevertebral segment of the left subclavian artery; however, owing to surgical difficulties to clearly visualize and manipulate the deeply located proximal left subclavian artery, the ligation was incomplete in two cases and impossible in the remaining case. It is also understandable that a redo operation in these cases is even more hazardous and by consequence, a minimally invasive alternative treatment is preferred. Persistent flow through the left subclavian artery was identified in all three cases by contrast-enhanced CT-scan, underlining the value of regular follow-up CT-scan after endovascular repair of aortic pathologies. In all three cases the proximal left subclavian artery was approached by puncture of the left brachial artery; the decision to occlude with coils [19-21] or plug [13,22-25] depended on the diameter of the prevertebral subclavian artery segment: if the segment was large enough for a plug (n = 2), then a plug was preferred owing to the ease of plug deployment; in the remaining case the prevertebral segment was too small for safe plug-deployment and microcoils were placed through a microcatheter. Except for a puncture site hematoma, no complications occurred during or after the procedure and in all cases no more perfusion of the occluded vessel was indentified on sequential follow-up CT-scan. The endovascular occlusion of the proximal left subclavian artery has been successfully performed in cases of intentional left subclavian artery coverage by the endograft, without previous carotid-subclavian transposition [13,19,20,22-25], using the same endovascular techniques. Finally, the gradual growth of the aneurismal sac or false lumen was stopped after the occlusion procedure.

## Conclusions

In summary, three cases of persistent flow through the left subclavian artery after combined open en endovascular surgery for thoracic aortic disease are presented. CT-scan clearly identified the persistent left subclavian artery opacification, despite previous surgical attempt of ligation; catheter-angiography confirmed these findings. Definitive occlusion of the prevertebral part of the left subclavian artery can be performed using plug or coils, resulting in disappearance of the endoleak and in cessation of the aneurismal or false lumen growth.

## Consent

In our institution no approval of the Ethical Committee is required for case reports.

## Competing interests

The authors declare that they have no competing interests.

## Authors' contributions

GM has taken care of the concept, design and the acquisition of data. SH as well as JV have taken care of the acquisition of data, the revision of the manuscript, and the final approval for the manuscript to be published. All contributing authors have read and approved the final manuscript.
